# Pertuzumab, trastuzumab, and chemotherapy in HER2-positive gastric/gastroesophageal junction cancer: end-of-study analysis of the JACOB phase III randomized clinical trial

**DOI:** 10.1007/s10120-022-01335-4

**Published:** 2022-09-06

**Authors:** Josep Tabernero, Paulo M. Hoff, Lin Shen, Atsushi Ohtsu, Manish A. Shah, Asna Siddiqui, Sarah Heeson, Astrid Kiermaier, Harrison Macharia, Eleonora Restuccia, Yoon-Koo Kang

**Affiliations:** 1 Medical Oncology Department, Vall d’Hebron Hospital Campus and Institute of Oncology (VHIO), UVic-UCC, IOB-Quirón, Passeig de la Vall d’Hebron 119-129, Barcelona, 08035 Spain; 2 IDOR, Hospital Vila Nova Star, Rede D’Or-Sao Luiz, Sao Paulo, Brazil; 3 Key Laboratory of Carcinogenesis and Translational Research (Ministry of Education/Beijing), Department of Gastrointestinal Oncology, Peking University Cancer Hospital and Institute, Haidian District, Beijing, People’s Republic of China; 4 Exploratory Oncology Research & Clinical Trial Center, National Cancer Center Hospital, Kashiwa, Japan; 5 Meyer Cancer Center at Weill Cornell Medical College, New York, NY USA; 6 Product Development Oncology, Roche Products Limited, Welwyn Garden City, UK; 7 Pharmaceutical Research and Early Development (pRED), F. Hoffmann-La Roche Ltd, Basel, Switzerland; 8 Biostatistics, F. Hoffmann-La Roche Ltd, Basel, Switzerland; 9 Product Development Oncology, F. Hoffmann-La Roche Ltd, Basel, Switzerland; 10 Department of Oncology, Asan Medical Center, University of Ulsan College of Medicine, Seoul, Korea

**Keywords:** HER2-positive, Gastric Cancers, Pertuzumab, Trastuzumab, Metastatic

## Abstract

**Background:**

Dual-targeted anti-HER2 therapy significantly improves outcomes in HER2-positive breast cancer and could be beneficial in other HER2-positive cancers. JACOB’s end-of study analyses aimed to evaluate the long-term efficacy and safety of pertuzumab plus trastuzumab and chemotherapy for previously untreated HER2-positive metastatic gastric or gastroesophageal junction cancer.

**Methods:**

Eligible patients were randomized 1:1 to pertuzumab/placebo plus trastuzumab and chemotherapy every 3 weeks. Primary endpoint: overall survival (OS). Secondary endpoints included progression-free survival (PFS), objective response rate (ORR), duration of response (DoR), and safety.

**Results:**

The intention-to-treat population comprised 388 patients in the pertuzumab arm and 392 in the placebo arm. The safety population comprised 385 and 388 patients, respectively. Median follow-up was ≥ 44.4 months. Median OS was increased by 3.9 months (hazard ratio 0.85 [95% confidence intervals, 0.72–0.99]) and median PFS by 1.3 months (hazard ratio 0.73 [95% confidence intervals, 0.62–0.85]) in the pertuzumab vs. the placebo arm. ORR was numerically higher (57.0% vs. 48.6%) and median DoR 1.8 months longer with pertuzumab treatment. There was a trend for more favorable hazard ratios in certain subgroups related to HER2 amplification/overexpression. Safety was comparable between arms, except for serious and grade 3–5 adverse events, and any-grade diarrhea, which were more frequent with pertuzumab.

**Conclusions:**

JACOB did not meet its primary endpoint. Nonetheless, the study continues to demonstrate some, albeit limited, evidence of treatment activity and an acceptable safety profile for pertuzumab plus trastuzumab and chemotherapy in previously untreated HER2-positive metastatic gastric or gastroesophageal junction cancer after long-term follow-up.

*Trial registration* NCT01774786; https://clinicaltrials.gov/ct2/show/NCT01774786.

## Introduction

The Trastuzumab for Gastric Cancer (ToGA) study demonstrated significantly improved overall survival (OS) with the addition of trastuzumab to chemotherapy in patients with previously untreated HER2-positive locally advanced/metastatic gastric/gastroesophageal junction cancer (GC/GEJC) [1]. Adding pertuzumab to trastuzumab and chemotherapy has been shown to significantly improve clinical outcomes in patients with HER2-positive early and metastatic breast cancer (BC) and the combination has been approved for patients with HER2-positive colorectal cancer in Japan based on the results of the TRIUMPH study [2–6]; however, there are several differences in tumor biology between BC and GC, including a higher incidence of HER2 heterogeneity and incomplete membrane staining in GC [7]. JACOB was designed to assess the efficacy and safety of pertuzumab plus trastuzumab and chemotherapy in patients with previously untreated HER2-positive metastatic GC/GEJC [6]. Primary results showed that addition of pertuzumab did not significantly improve OS at  ≥ 24.4 months median follow-up [8]. We report descriptive end-of-study results, including previously unreported biomarker results, at  ≥ 44.4 months median follow-up.

## Methods

### Study design and patients

JACOB (NCT01774786) was a double-blind, placebo-controlled, randomized, multicenter, phase III trial. Details have been published previously [8].

Eligible patients with previously untreated HER2-positive (centrally assessed immunohistochemistry [IHC] 3  + or IHC 2  +  /in situ hybridization [ISH]-positive) GC/GEJC (*n* = 780) were randomized 1:1 to intravenous pertuzumab (840 mg) / placebo plus trastuzumab (8 mg/kg loading, 6 mg/kg maintenance doses) and chemotherapy (intravenous cisplatin 80 mg/m^2^ plus capecitabine 1000 mg/m^2^ orally twice daily, or intravenous 5-flurouracil 800 mg/m^2^ every 24 h continuously for 120 h) every 3 weeks. Chemotherapy discontinuation during/before cycle 6 was allowed for progressive disease/unacceptable toxicity. Chemotherapy continuation post-cycle 6 was at the discretion of the patient and treating physician. Pertuzumab/placebo and trastuzumab were continued following chemotherapy completion until disease progression, unacceptable toxicity, or withdrawal from the study.

The primary endpoint was OS. Secondary endpoints included progression-free survival (PFS), objective response rate (ORR), duration of response (DoR), and safety. Exploratory endpoints included association of biomarkers with efficacy outcomes.

To assess HER2 heterogeneity further, subgroups were defined by percentages of stained cancer cells, per the following categories: focal staining: 0–29% of cells staining positive for HER2; heterogenous staining: 30–79% of cells staining positive for HER2; and homogenous staining: 80–100% of cells staining positive for HER2. Subgroups were also defined by the gene copy number; this term refers to the number of copies of the *HER2* gene that were determined by an in situ hybridization test. An average of 6 copies of the *HER2* gene per cell/nucleus was used as a cutoff for this analysis to signify gene amplification. Per the American Society of Clinical Oncology (ASCO) guidelines, a gene copy number of > 6 signifies true amplification, whereas 4–6 copies is considered an equivocal result [9].

### Statistical analysis

Target sample sizes and power calculations for the primary analysis have been reported [8]. Analyses were performed with SAS (version 9.2 and 9.4; SAS Institute Inc., NC, USA). OS and PFS were analyzed in the intention-to-treat (ITT) population (all randomized patients regardless of whether they received study treatment) according to study arm allocation. ORR was assessed in patients in the ITT population with measurable disease at baseline; those with no tumor assessment data after baseline were classed as non-responders. Safety analyses were conducted in all patients who received at least one dose of study treatment and was analyzed according to arm allocation. The Kaplan–Meier method was used to estimate OS, PFS, and DoR, with 95% confidence intervals (CIs) calculated using the Brookmeyer–Crowley method. A stratified Cox proportional hazards regression model was used to estimate the hazard ratio (HR) between arms with 95% CIs (stratification factors: geographic region, HER2 status, previous gastrectomy). The proportion of patients who achieved an overall response was summarized and 95% CIs calculated using the Clopper–Pearson method. Adverse events (AEs) were graded per standard criteria. All analyses are descriptive.

## Results

### Patients

**Fig. 1:**
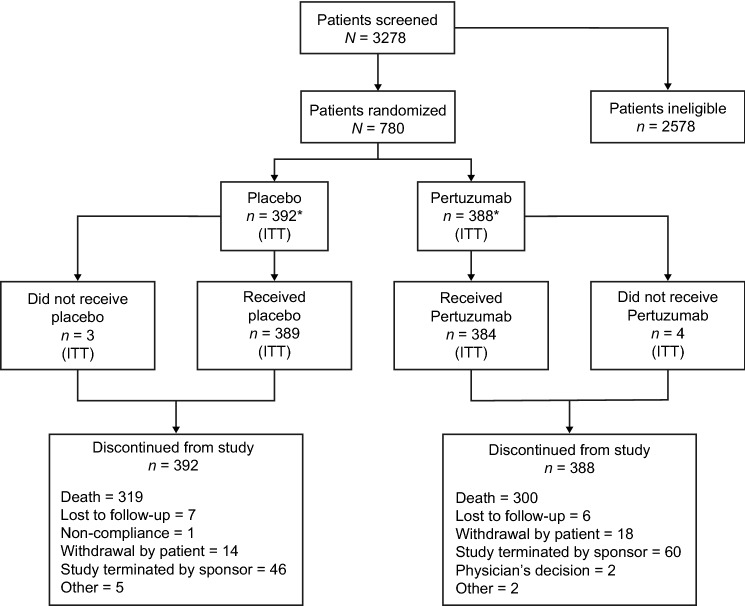
Patient disposition. *ITT* intention-to-treat. *One patient assigned to the placebo group received one dose of pertuzumab in error and was included in the safety population for the pertuzumab group; final safety population: pertuzumab group (*n* = 385), placebo group (*n* = 388)

Of the 780 eligible patients enrolled between June 10, 2013 and January 12, 2016, 388 were randomized to the pertuzumab arm and 392 to the placebo arm (ITT population) [8]. The safety population comprised 385 and 388 patients in the pertuzumab and placebo arms, respectively (including one who received both treatments in error and was counted in the pertuzumab arm) [8]. Patient dispositions are shown in Fig. 1. The clinical cutoff for this analysis was January 24, 2020.

### Efficacy

**Fig. 2:**
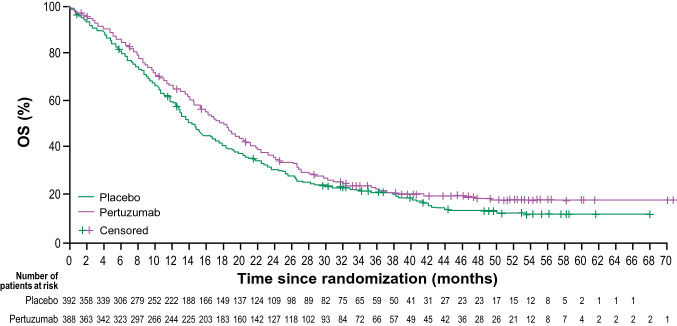
OS in the ITT population (primary endpoint follow-up). *ITT* intention-to-treat, *OS* overall survival

At a median follow-up of 46.1 and 44.4 months, respectively, there were 300 OS and 342 PFS events in the pertuzumab arm, and 319 OS and 353 PFS events in the placebo arm). Median OS increased by 3.9 months in the pertuzumab vs. placebo arm; a 15% reduction in risk of death (HR 0.85 [95% CI, 0.72–0.99]) (Table 1 and Fig. 2). Median PFS increased by 1.3 months in the pertuzumab vs. placebo arm; a 27% reduction in risk of progressive disease (HR 0.73 [95% CI, 0.62–0.85]) (Table 1). ORR was numerically higher (57.0% vs. 48.6%) and median DoR was 1.8 months longer in the pertuzumab vs. placebo arm (Table 1).

**Table 1:** Efficacy summary (ITT population)

	Pertuzumab + trastuzumab + chemotherapy (*n* = 388)	Placebo + trastuzumab + chemotherapy (*n* = 392)
*Primary endpoint follow-up*		
OS events, *n*	300	319
Median OS, months (95% CI)	18.1 (16.2–19.5)	14.2 (12.9–15.7)
Stratified HR (95% CI)	0.85 (0.72–0.99)	
Median duration of OS follow-up, months	46.1	44.4
*Secondary endpoints*		
PFS events, *n*	342	353
Median PFS, months (95% CI)	8.5 (8.3–9.7)	7.2 (6.4–8.2)
Stratified HR (95% CI)	0.73 (0.62–0.85)	
Median duration of PFS follow-up, months	50.4	47.4

	*n* = 351	*n* = 352
ORR, *n* (%)^a^		
*Responders*	200 (57.0)	171 (48.6)
Complete response	20 (5.7)	7 (2.0)
Partial response	180 (51.3)	164 (46.6)
*Non-responders*	151 (43.0)	181 (51.4)
Stable disease	97 (27.6)	115 (32.7)
Progressive disease	17 (4.8)	29 (8.2)
Missing assessment	37 (10.5)	37 (10.5)

	*n* = 203	*n* = 175
Median DoR, months (95% CI)	10.2 (8.5–12.0)	8.4 (6.8–9.1)

^a^ORR was assessed in patients in the ITT population with measurable disease at baseline; those with no tumor assessment data after baseline were classed as non-responders

*CI* confidence interval, *DoR* duration of response, *HR* hazard ratio*, ITT* intention to treat*, ORR* objective response rate*, OS* overall survival*, PFS* progression-free survival

### OS subgroup analyses

**Fig. 3:**
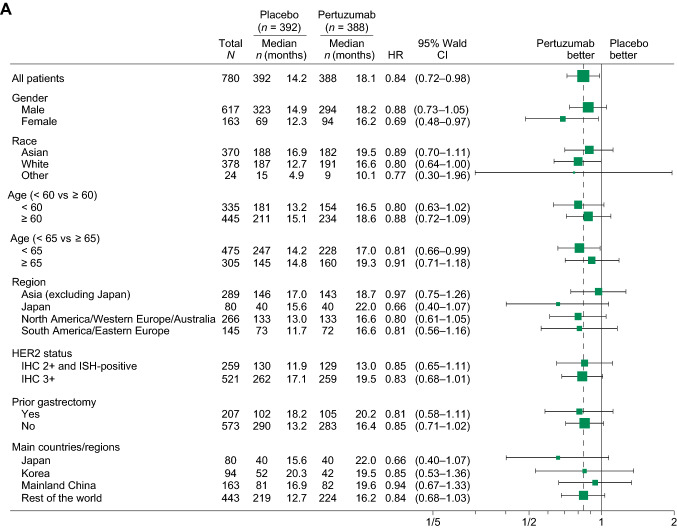

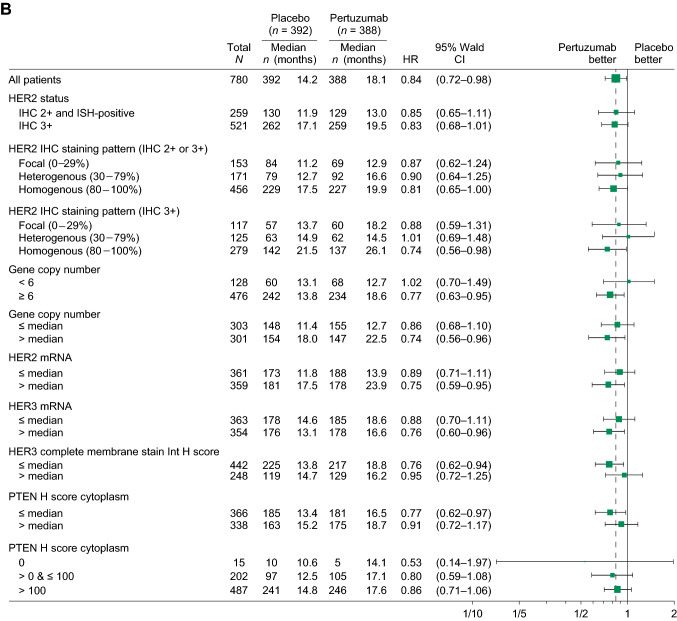
OS by biomarker subgroups (ITT population). *CI* confidence interval*, HER2* human epidermal growth factor receptor 2*, HER3* human epidermal growth factor receptor 3, *HR* hazard ratio*, H score* histologic score*, IHC* immunohistochemistry*, ISH* in situ hybridization*, ITT* intention to treat*, OS* overall survival*, PTEN* phosphatase and tensin homolog. **A** OS by baseline characteristics and stratification factors. **B** OS by biomarker subgroups

When OS was analyzed by baseline characteristics and stratification factors, HRs were similar to that observed in the ITT population for most subgroups (Fig. 3A). Similarly, no clear differences in HR were observed in biomarker subgroups, although there was a trend for more favorable HRs in certain subgroups related to HER2 amplification/overexpression (Fig. 3B). OS was longer for patients with a HER2 status of IHC 3  + vs. IHC 2  +  /ISH-positive; with homogenous HER2 IHC staining patterns vs. heterogenous or focal staining patterns; and with higher *HER2* copy numbers/mRNA levels in both arms (Fig. 3B). HER3 and phosphatase and tensin homolog (PTEN) did not show any association with OS (Fig. 3B).

### Safety

Safety is summarized in Table 2. Incidences of any-grade AEs, fatal AEs, AEs that led to dose modifications, and cardiac AEs were similar between treatment arms. Incidence of symptomatic and asymptomatic left ventricular systolic dysfunction was low. Serious AEs, grade 3–5 AEs, and any-grade diarrhea were more frequent in the pertuzumab arm. Most diarrhea events were grade 1 or 2.

**Table 2:** Safety summary (safety population)

No. of patients with ≥ 1 AE (%)^a^	Pertuzumab + trastuzumab + chemotherapy (*n* = 385)	Placebo + trastuzumab + chemotherapy (*n* = 388)
*Overall safety*		
Any grade AE	381 (99.0)	385 (99.2)
Any grade diarrhea	241 (62.6)	139 (35.8)
AE with fatal outcome	27 (7.0)	31 (8.0)
Serious AE	178 (46.2)	156 (40.2)
Grade 3–5 AE	310 (80.5)	288 (74.2)
*Dose modifications*		
AE leading to pertuzumab/placebo discontinuation	48 (12.5)	46 (11.9)
AE leading to pertuzumab/placebo interruption and/or delay	110 (28.6)	94 (24.2)
*Cardiac safety*		
Symptomatic LVSD	3 (0.8)	1 (0.3)
Asymptomatic LVSD	20 (5.2)	18 (4.6)

^a^Multiple occurrences of the same AE in one individual are counted only once

*AE* adverse event, *LVSD* left ventricular systolic dysfunction

## Discussion

Although JACOB failed to meet its primary endpoint [8], these end-of-study analyses confirm some evidence of activity. Median OS was 18.1 months in the pertuzumab arm and 14.2 months in the placebo arm at ≥ 44.4 months’ median follow-up (stratified HR 0.85 [95% CI, 0.72–0.99]), signifying a 15% reduction in risk of death when adding pertuzumab to trastuzumab and chemotherapy in patients with previously untreated metastatic GC/GEJC. At the primary analysis, the OS HR was 0.84 (95% CI, 0.71–1.00; *p* = 0.057); there were 242 and 262 OS events in the pertuzumab and placebo arms, respectively. Compared with the primary analysis, there was an increase in number of OS events and a decrease in the CI size in this end-of study analysis, thus supporting that the end-of-study analysis has increased statistical power when compared with the primary analysis.

The overall safety profile was acceptable and comparable between arms; however, the incidence of serious AEs, grade 3–5 AEs, and any-grade diarrhea was higher in the pertuzumab arm than in the placebo arm. There was no increase in the number of patients who experienced any-grade AEs; however, there was a slight increase in the number of patients who experienced serious AEs (final analysis: 45% and 39%; end-of-study analysis: 46.2% and 40.2%) and grade 3–5 AEs (final analysis: 80% and 73%; end-of-study analysis: 80.5% and 74.2%) in both the pertuzumab and placebo arms, respectively. Results from OS baseline characteristic and stratification factor subgroup analyses were consistent with those of the primary analysis [8]. Overall, biomarker analyses did not reveal a clear and consistent predictive effect for any of the markers tested. Consistent with biomarker results from GATSBY [10], increased OS was observed in both arms in patients with elevated or more homogeneously expressed HER2 levels (although this was not tested statistically). This suggests that gastric tumors with high and uniform HER2 expression may be more driven by HER2 signaling, and are more sensitive to anti-HER2 therapy. PTEN protein expression levels did not seem to affect OS. Total loss of PTEN protein, which is hypothesized to contribute to resistance to HER2-targeted therapy, could not be evaluated due to small sample size. The study design included HER2-targeted therapy in the control arm, which may limit detection of biomarker signals in this analysis. Therefore, only markers that produced large effects may have been detectable, while smaller effects may have been masked.

The limited treatment effect of combining pertuzumab with trastuzumab and chemotherapy in GC may be multi-factorial. Underlying differences in HER2 expression between GC and BC and the increased complexity of GC suggests that HER2 signaling may not be the only driver of disease progression in some patients [7]. Lapatinib, an anti-HER2 tyrosine kinase inhibitor, also failed to show improved OS as a first-line treatment in combination with capecitabine and oxaliplatin in patients with HER2-positive metastatic GC, esophageal, or gastroesophageal adenocarcinoma [11]. Similarly, trials of anti-HER2 therapies (trastuzumab, lapatinib, and ado-trastuzumab emtansine) in patients with previously treated HER2-positive metastatic GC have also failed to show improved OS [12–14]. Therefore, success in advancing treatment for HER2-positive metastatic GC has been limited to the advent of trastuzumab in combination with chemotherapy, which has remained the first-line standard of care for a decade. Recently, the U.S. Food and Drug Administration (FDA) granted accelerated approval for the use of pembrolizumab in combination with first-line trastuzumab and chemotherapy for the treatment of patients with HER2-positive metastatic GC [15], based on the interim data from the ongoing KEYNOTE-811 study (NCT03615326) [16]. In this study, pembrolizumab, trastuzumab, and chemotherapy showed significantly improved ORR vs. placebo, trastuzumab, and chemotherapy (74% vs. 52%) and a manageable toxicity profile, with similar incidence of AEs across arms; the median DoR was 10.6 months in the pembrolizumab arm and 9.5 months in the placebo arm [16]. In previously treated patients, trastuzumab deruxtecan demonstrated superior efficacy (ORR and OS) vs. physician’s choice of either irinotecan or paclitaxel monotherapy, becoming a new standard of care in the US [17]. There remains an unmet medical need in HER2-positive GC/GEJC, and a wide array of novel therapies and treatment regimens are currently under investigation in these patients; these include anti-HER2 monoclonal antibodies (margetuximab), checkpoint inhibitors (nivolumab and ipilimumab), tyrosine kinase inhibitors (tucatinib), and bispecific antibodies that target HER2 (zanidatamab) [18–22]. There have also been several developments in screening techniques, such as next-generation sequencing and liquid biopsy, which can supplement the traditional diagnostic tests to further understand the complexity of HER2-positive GC and identify targetable genomic alterations [23, 24]. In addition, multiple screening techniques (composite testing) or multiple samples taken from different sites or lesions can be used to assess HER2 heterogeneity within tumors, which can cause discordant results regarding the HER2 status between biopsies and could be associated with disease progression, to provide an accurate assessment of a patient’s HER2 status and allow selection of an optimal treatment regimen [25].

Limitations of the study include the lack of power for some of the subgroup analyses, and the resulting large, overlapping CIs.

## Conclusions

Although JACOB did not meet its primary endpoint, the study continues to demonstrate some, albeit limited, evidence of treatment activity and an acceptable safety profile for pertuzumab plus trastuzumab and chemotherapy in previously untreated HER2-positive metastatic GC/GEJC after long-term follow-up (≥ 44.4 months)**.**

## Data Availability

Qualified researchers may request access to individual patient-level data through the clinical study data request platform: https://vivli.org/. Further details on Roche's criteria for eligible studies are available here: https://vivli.org/members/ourmembers/. For further details on Roche's Global Policy on the Sharing of Clinical Information and how to request access to related clinical study documents, see here: https://www.roche.com/research_and_development/who_we_are_how_we_work/clinical_trials/our_commitment_to_data_sharing.htm.
